# Study of Five Pubertal Transition-Related Gene Polymorphisms as Risk Factors for Premature Coronary Artery Disease in a Chinese Han Population

**DOI:** 10.1371/journal.pone.0136496

**Published:** 2015-08-25

**Authors:** Bin Chen, Fangyi Xie, Chengchun Tang, Genshan Ma, Li Wei, Zhong Chen

**Affiliations:** 1 Department of Cardiology, Shanghai Jiao Tong University Affiliated Sixth People’s Hospital, No. 600 Yishan Road, Shanghai 200233, China; 2 Central Laboratory, Shanghai Jiao Tong University Affiliated Sixth People’s Hospital, No. 600 Yishan Road, Shanghai 200233, China; 3 Department of Cardiology, The Affiliated Zhongda Hospital of Southeast University, No. 87 Dingjiaqiao, Nanjing 210009, China; 4 Department of Endocrinology, Shanghai Jiao Tong University Affiliated Sixth People’s Hospital, No. 600 Yishan Road, Shanghai 200233, China; CSIR-INSTITUTE OF GENOMICS AND INTEGRATIVE BIOLOGY, INDIA

## Abstract

**Background:**

Recently, single nucleotide polymorphisms (SNPs) (DLK-rs10144321, SIX6-rs1254337, MKRN3-rs12148769, LIN28B-rs7759938, and KCNK9-rs1469039) were found to be strongly associated with age at menarche. Recent studies also suggested that age at menarche is a heritable trait and is associated with risks for obesity, type 2 diabetes mellitus (T2DM), cardiovascular disease, and all-cause mortality. Since an association between these five SNPs and premature coronary artery disease (CAD) has never been reported, we investigated whether these SNPs are associated with premature CAD and its severity in a Chinese Han population.

**Methods:**

We enrolled 432 consecutive patients including 198 with premature CAD (<55 years in men and <65 years in women) and 234 controls. All subjects were genotyped for the five SNPs by the PCR-ligase detection reaction method. The associations between these SNPs and premature CAD and its severity were analyzed.

**Results:**

The following genotypes were identified: GG, AG, and AA at rs10144321 and rs12148769; TT, AT, and AA at rs1254337; CC, CT, and TT at rs1469039; and TT and CT at rs7759938. Significant differences in genotype distribution frequencies at rs1254337 were found between controls and patients with premature CAD (*P*<0.05). No associations were found between the five SNPs and the severity of coronary lesions (all *P*>0.05). Compared with controls, patients with premature CAD had a higher prevalence of T2DM and dyslipidemia, and the proportion of patients with T2DM rose significantly with an increase in the number of stenosed coronary vessels (all *P*<0.05). After adjustment for the clinical parameters in multivariable analysis, three factors were identified that significantly increased the risk of premature CAD: the AA genotype at rs1254337 (OR: 2.388, 95% CI: 1.190–4.792, *P* = 0.014), male gender (OR: 1.565, 95% CI: 1.012–2.420, *P* = 0.044), and T2DM (OR 2.252, 95% CI: 1.233–4.348, *P* = 0.015).

**Conclusions:**

Among the five pubertal transition-related gene polymorphisms, we identified an association between rs1254337 and premature CAD in a Chinese Han population.

## Background

The incidence of death from coronary artery disease (CAD) and acute myocardial infarction increased from 2002 to 2011 in China. In 2002, the mortality from CAD was 39.6 and 27.6 per 100,000 in urban and rural areas, respectively; and this increased to 96.0 and 75.7 per 100,000 in 2011 [1]. Genetic background plays an important role in the etiology of CAD [2–5], and CAD is a complex disease stemming from interactions between multiple genetic and environmental risk factors. Therefore, there is growing interest in finding novel genetic markers to improve risk stratification, and the early detection and treatment of high-risk patients has emerged as a key strategy in the prevention of cardiovascular disease (CVD) [6].

The prevalence of CVD is associated with socioeconomic prosperity, lifestyle changes, advancing age, and urbanization [7]. Patients with traditional risk factors, such as obesity, cigarette smoking, type 2 diabetes mellitus (T2DM), hyperlipidemia, and a family history of CAD, are at a higher risk for CAD, future cardiac death, and worse outcomes [1,8–10]. Furthermore, the morbidity and mortality from CAD in patients with premature CAD (males <55 years and females <65 years) may have a devastating impact on the families of these patients [11].

Age at menarche acts as a marker of puberty timing in females, and recent studies suggest that this age is a heritable trait, and early pubertal onset is associated with increased risks for elevated weight and/or body mass index (BMI), obesity, T2DM, CVD, cancer, and all-cause mortality [12–15]. More recently, Perry et al. [14] used genome-wide and custom-genotyping arrays in 182,416 women of European descent and found 123 signals at 106 genomic loci associated with age at menarche (P<5×10^−8^). Among these interesting loci, five single nucleotide polymorphisms (SNPs) (DLK-rs10144321, SIX6-rs1254337, MKRN3-rs12148769, LIN28B-rs7759938, and KCNK9-rs1469039) were identified that belong to the forest plot of parent-of-origin-specific allelic associations at three imprinted menarche loci. Taken together, these novel findings suggest that these five SNPs might be associated with an increased risk of premature CAD and its severity. Genetic markers of CAD should facilitate the development of effective preventive strategies to attenuate the rapid rise in morbidity and mortality from CAD.

However, to date, no information has been published on the potential association of these five SNPs with the risk of premature CAD. Therefore, the aim of the present study was to analyze, for the first time, whether these five SNPs are associated with premature CAD and its severity in a Chinese Han population.

## Methods

### Study subjects

We enrolled 432 unrelated patients of Han ethnicity who underwent elective coronary angiography (CAG) for suspicion of CAD from January 2008 to December 2010. The subjects included 198 patients (men aged 38~54 years and women aged 40~64 years) with premature CAD and 234 controls. Among these subjects, at least three generations had resided in China. CAD was diagnosed as stenosis ≥ 50% in at least one of the three main coronary arteries or major branches and a history of myocardial infarction defined by World Health Organization criteria, or a history of coronary balloon angioplasty, coronary stent implantation, or coronary artery bypass surgery. Premature CAD was defined as CAD diagnosed in males before 55 years old and in females before 65 years old. The mean duration of CAD in the 198 patients with premature CAD was 1.3 years. There were 234 participants without detectable coronary stenosis or elevated cardiac troponin I levels who served as controls. The exclusion criteria included the following: autoimmune disease, severe liver or kidney disease, a contraindication for heparin use, or a history of childhood adversity (physical abuse, sexual abuse, neglect, biological father absent from the home, other parent loss, parental mental illness, parental substance abuse, parental criminality, interparental violence, serious physical illness in childhood and family economic adversity). The Medical Research Ethics Committee of the Affiliated Zhongda Hospital of Southeast University reviewed and approved the present study. Written informed consent was obtained from each participant before the study began.

### Elective coronary angiography

The methods used for coronary angiography (CAG) and grading the severity of coronary stenosis were described previously [3]. Based on the CAG results, the premature CAD patients were assigned to one of three groups according to the number of stenosed coronary arteries (1-, 2- and 3-vessel disease).

### Data collection and definition of cardiovascular risk factors

A physical examination including anthropometric parameters and blood testing was performed on each subject by a qualified doctor using standard methods. Height assessment was accurate to 0.1 cm, weight was measured to an accuracy of 0.1 kg, and body mass index (BMI) was calculated as previously reported [5]. Each patient was evaluated for CAD risk factors, such as hypertension, T2DM, and smoking habits.

Plasma levels of fasting blood sugar (FBS), total cholesterol (TC), triglyceride (TG), high-density lipoprotein cholesterol (HDL-C), low-density lipoprotein cholesterol (LDL-C), apolipoprotein A1 (apo A1), apolipoprotein B (apo B), and lipoprotein (Lp)(a)were measured by standard enzymatic methods. TG levels were measured directly when >4.52 mmol/L (400 mg/dL). Dyslipidemia was defined as a TG level ≥1.70 mmol/L, TC ≥5.72 mmol/L, or HDL-C <0.90 mmol/L in males or <1.04 mmol/L in females.

### DNA extraction and genotyping

DNA was extracted using a method that was described previously [3]. Genotyping for the five SNPs (rs10144321, rs1254337, rs12148769, rs7759938, and rs1469039) was performed by the modified method of polymerase chain reaction-ligase detection reaction as previously reported [16,17] using TaqMan genotyping assays on an ABI Prism 3730 Sequence Detection System (Applied Biosystems, Foster City, CA).

### Statistical analyses

Statistical analyses were done using SPSS 19.0 software (SPSS, Inc, Chicago, IL). A Student’s *t* test, one-way ANOVA, or chi-square test was used as appropriate. The χ2 goodness of fit for the Hardy-Weinberg equilibrium of the five SNPs among premature CAD cases and controls were calculated separately. Multivariable logistic regression analyses were used to calculate odds ratios (OR) [with 95% confidence intervals (CI)]. A chi-square test was used to compare the distribution frequencies of genotypes at the five SNPs between controls and patients with premature CAD, or among different groups of CAD patients according to the number of stenosed coronary arteries. All *P*-values were two-tailed with a significance level of 0.05.

## Results

### Analyses of baseline characteristics in the premature CAD and control groups (Table 1)

**Table 1:** Baseline data of the study population.

	Controls	Premature CAD	*P*
**Numbers, n**	234	198	
**Sex**			
**male, n (%)**	105 (44.87)	104 (52.52)	0.113
**female, n (%)**	129 (55.13)	94 (47.48)	0.113
**Age, years**	52.34±5.60	52.22±5.95	0.741
**Hypertension, n (%)**	135 (57.69)	125 (63.13)	0.250
**Type 2 diabetes mellitus, n (%)**	25 (10.68)	39 (19.70)	0.009
**Dyslipidemia, n (%)**	67 (28.63)	77 (38.89)	0.024
**Smokers, n (%)**	68 (29.06)	54 (27.27)	0.681
**BMI (kg/m** ^**2**^ **)**	24.48±3.17	25.38±4.11	0.847
**Fasting blood sugar (mmol/L)**	5.28±1.33	5.54±1.55	0.110
**TC (mmol/L)**	3.71±1.67	3.78±1.49	0.685
**TG (mmol/L)**	2.27±1.52	2.69±1.74	0.026
**LDL-C (mmol/L)**	2.74±0.73	2.83±0.75	0.293
**HDL-C (mmol/L)**	1.17±0.26	1.16±0.28	0.836
**Apo A1 (g/L)**	1.18±0.24	1.16±0.21	0.350
**Apo B (g/L)**	0.84±0.27	0.92±0.27	0.010
**Lp(a) (mg/dL)**	197.35±140.41	216.66±161.98	0.498

Apo A1, apolipoprotein A1; Apo B, apolipoprotein B; BMI, body mass index; CAD, coronary artery disease; HDL-C, high-density lipoprotein-cholesterol; LDL-C, low-density lipoprotein-cholesterol; Lp(a), lipoprotein(a); TC, total cholesterol; TG, triglyceride.

Patients with premature CAD had higher concentrations of TG and apo B, and a higher prevalence of T2DM and dyslipidemia compared with controls (all *P*<0.05). However, the mean values of age, BMI, FBS, TC, LDL-C, HDL-C, apo A1 and Lp(a) did not differ significantly between the two groups. Furthermore, there were no significant differences in the distribution of gender and smokers between the two groups (all *P*>0.05).

### Comparison of the clinical characteristics among the control and CAD patients grouped according to the number of diseased coronary arteries (Table 2)

When all participants were divided into four groups that included controls and premature CAD patients with 1-, 2-, and 3-vessel disease, the proportion of patients with T2DM rose significantly with the increment in the number of stenosed coronary vessels (*P* = 0.033). There were no significant differences in the distribution of gender, hypertension, dyslipidemia, and smokers among the four groups (all *P*>0.05) (Table 2).

**Table 2:** Comparison of the clinical characteristics among the controls and CAD patients grouped according to the number of diseased coronary arteries.

		Premature CAD (n = 198), number of vessels involved			
Variants	Controls (n = 234)	one (n = 105)	two (n = 46)	three (n = 47)	*P* values
**Sex (male), n**	105	51	25	28	0.244
**Hypertension, n**	135	63	29	33	0.434
**Type 2 diabetes mellitus, n**	25	18	9	12	0.033
**Dyslipidemia, n**	67	41	15	21	0.086
**Smokers, n**	68	23	16	15	0.328

*P* is the significance level of comparison of clinical characteristics among 4 groups by a chi-square test. CAD, coronary artery disease.

### Polymorphism frequency distribution between the premature CAD and control groups (Table 3)

The following genotypes were identified: GG, AG, and AA at rs10144321 and rs12148769; TT, AT, and AA at rs1254337; CC, CT, and TT at rs1469039; TT and CT at rs7759938; and CC, CG, and GG at rs4808793. No deviation from the Hardy-Weinberg equilibrium was detected in the control and premature CAD groups. Table 3 shows the results of evaluation of the frequencies of genotypes for the five polymorphisms between the control and premature CAD groups.

**Table 3:** Genotype distributions at the five SNPs in the control and premature CAD groups.

	Control, n(%)	Premature CAD, n(%)	OR (95% CI)
**rs10144321**			
**GG**	73(31.2) *	62 (31.3)*	1.0 (reference)
**AG**	104(44.4)	100(50.5)	0.883 (0.571–1.366)
**AA**	57 (24.4)	36 (18.2)	1.345 (0.786–2.301)
***P***	0.255		
**rs1254337**			
**TT**	124(53.0)	81 (40.9)	1.0 (reference)
**AT**	92 (39.3)	90 (45.5)	1.498 (1.000–2.242)
**AA**	18 (7.7)	27 (13.6)	2.296 (1.188–4.438)#
***P***	0.019		
**rs12148769**			
**GG**	94 (40.2)	84 (42.4	1.0 (reference)
**AG**	114 (48.7)	94 (47.5)	1.084 (0.725–1.619)
**AA**	26 (11.1)	20 (10.1)	1.162 (0.605–2.232)
***P***	0.874		
**rs7759938**			
**TT**	227 (97.0)	185 (93.4)	1.0 (reference)
**CT**	7 (3.0)	13 (6.6)	2.279(0.891–5.829)
***P***	0.078		
**rs1469039**			
**CC**	102 (43.6)	99 (50.0)	1.0 (reference)
**CT**	105 (44.9)	87 (43.9)	1.864(0.892–3.896)
**TT**	27 (11.5)	12 (6.1)	1.184(0.748–1.550)
***P***	0.104		

*Number of individuals with percentage in parentheses. CAD, coronary artery disease; OR, odds ratio; CI, confidence intervals. The chi-square test and likelihood ratio test were used to analyze the genotypes. *P* is the level of significance for the premature CAD group compared with the control group.

^#^ *P* = 0.013 compared with the TT genotype.

Significant differences were found in the genotype distribution frequencies at rs1254337 between controls and patients with premature CAD (*P* = 0.019). Binary logistic regression analysis revealed an association between rs1254337 and premature CAD. Compared with the TT genotype, patients with the AA genotype had a higher risk of premature CAD (OR: 2.296, 95% CI: 1.188–4.438, *P* = 0.013). However, there were no associations between premature CAD and the frequencies of the genotypes at rs10144321, rs12148769, rs7759938, and rs1469039.

Further analysis showed there were no significant differences among the three genotypes at rs1254337 and the average age, or the mean values of FBS, TC, TG, LDL-C, HDL-C, apo A1, apo B, LP(a), and BMI. In addition, there were no significant differences in the distribution of gender, hypertension, T2DM, dyslipidemia, and smokers among the three genotypes at rs1254337 (all *P*>0.05).

### Distribution of genotypes at the five SNPs in CAD patients grouped according to the severity of coronary lesions (Table 4)

When all patients with premature CAD were grouped according to the number of stenosed vessels (1-, 2-, and 3-vessel disease), there were no significant differences in the distribution of the genotypes at the five SNPs among the three groups (all *P*>0.05) (Table 4).

**Table 4:** Distribution of genotypes at the five SNPs in CAD patients grouped according to the severity of coronary lesions.

		Number of vessels involved			
Variants	Genotypes	One	Two	Three	*P* values
**rs10144321**	GG, n	33	16	13	
	AG, n	54	21	25	0.937
	AA, n	18	9	9	
**rs1254337**	TT, n	40	19	22	
	AT, n	49	20	21	0.759
	AA, n	16	7	4	
**rs12148769**	GG, n	48	18	18	
	AG, n	48	21	25	0.635
	AA, n	9	7	4	
**rs7759938**	TT, n	97	44	44	0.755
	CT, n	8	2	3	
**rs1469039**	CC, n	51	22	26	
	CT, n	47	21	19	0.930
	TT, n	7	3	2	

*P* is the significance level of comparison among three different groups according to the number of coronary arteries involved in patients with premature CAD. SNPs, single nucleotide polymorphisms.

### Multivariable logistic regression analysis of risk factors and different genotypes for the occurrence of premature CAD (Table 5)

Table 5 shows that after adjustment for the clinical parameters (smoking, hypertension, dyslipidemia, and continuous variables), three factors were identified that significantly increased the risk of premature CAD: the AA genotype at rs1254337 (OR: 2.388, 95% CI: 1.190–4.792, *P* = 0.014), male gender (OR: 1.565, 95% CI: 1.012–2.420, *P* = 0.044), and T2DM (OR 2.252, 95% CI: 1.233–4.348, *P* = 0.015).

**Table 5:** Multivariable logistic regression analysis of cardiovascular risk factors and different genotypes for the occurrence of premature CAD.

Variants		B	P	OR	95% CI
**rs1254337**	**TT**		0.000	1.00 (reference)	
	**AT**	0.310	0.156	1.364	0.888–2.093
	**AA**	0.870	0.014	2.388	1.190–4.792
**Sex, male**		0.448	0.044	1.565	1.012–2.420
**Type 2 diabetes mellitus**		0.843	0.015	2.252	1.233~4.348

See Table 3 for abbreviations.

## Discussion

This is the first case-control study assessing the relationship among five pubertal transition-related gene SNPs (rs10144321, rs1254337, rs12148769, rs7759938, and rs1469039), and the development and severity of premature CAD. The current study for the first time demonstrates an association between rs1254337 and premature CAD in a Chinese Han population. Compared with rs1254337 TT genotype carriers, the AA genotype carriers had a 1.388-fold higher risk of developing premature CAD. However, the current study was unable to provide supportive genetic prediction values for the other 4 SNPs and premature CAD.

Age at menarche acts as a marker of timing of puberty in females, and recent studies suggest that it is a heritable trait, and early pubertal onset is associated with an increased risk of obesity, T2DM, CVD, cancer, and all-cause mortality [12–15]. Pathway analyses implicated nuclear hormone receptors, particularly retinoic acid andγ-aminobutyric acid-B2 receptor signaling, as potential novel mechanisms that regulate pubertal timing in humans [14]. The presence of menarche age-raising alleles at rs1254337 was associated with lower adult BMI and taller adult height [14]. Epidemiological evidence suggests that early menarche (onset of menses ≤ 11 years) has increased in prevalence in recent birth cohorts and is associated with multiple unfavorable medical and mental health outcomes in adulthood. Available data support the concept that childhood adversities occurring prior to menarche contribute to early menarche. Childhood adversity predicts central obesity beyond more contemporary, modifiable risk factors [18]. In particular, childhood sexual abuse is the adversity most strongly associated with early menarche [19]. In the present study, we excluded participants who experienced childhood adversities or abuse to minimize the potential influence of these variables on the occurrence of premature CAD in adults.

In the present study, patients with premature CAD had higher concentrations of TG and apo B, and a higher prevalence of T2DM and dyslipidemia compared with controls. Furthermore, the proportion of patients with T2DM rose significantly with an increase in the number of stenosed coronary vessels. Hypercholesterolemia and T2DM are well-known risk factors for CAD, and investigators used to believe that familial combined hyperlipidemia (FCHL) carried a greater risk of premature CAD than familial hypertriglyceridemia (FHTG). However, recent clinical studies [20,21] have shown that FCHL and FHTG are more alike than dissimilar, and the risk of CAD in FCHL and FHTG is strongly related to the presence of metabolic syndrome. Elevated TG concentrations (as a feature of metabolic syndrome) are associated with an increased risk of premature CAD, and the association between the plasma TG levels and premature familial CAD is strong, graded, and independent. Apo B levels improve risk assessment of future CAD events above and beyond LDL-C or non-HDL-C levels [22], and non-HDL-C and apo B levels are each associated with the risk of future major cardiovascular events [23]. In conjunction with available data, these findings suggest that the hypertriglyceridemia is not benign and warrants aggressive treatment.

Based on our assessment of the relationships between these five pubertal transition-related gene polymorphisms and premature CAD and its severity, we found a significant difference in the genotype distribution frequencies at rs1254337 between controls and premature CAD patients. Furthermore, binary logistic regression analysis revealed an association of the rs1254337 polymorphism with premature CAD; patients with the AA genotype had a 1.388-fold higher risk of premature CAD compared with GG genotype carriers. Moreover, multivariable logistic regression analysis showed that the association between the AA genotype and premature CAD was independent of other clinical variables. However, we could not find any significant relationships between rs1254337 and the clinical and metabolic characteristics of our study subjects. Also, we did not detect any significant differences in the genotype frequency distributions of these five SNPs among CAD patients with 1-, 2-, and 3-vessel disease. In addition, there were no significant differences in the frequencies of genotypes at rs10144321, rs12148769, rs7759938, and rs1469039 between controls and patients with premature CAD.

Considering the known effects of lipid disorders on premature CAD [20–23], it was surprising that we did not detect any differences in the clinical or metabolic characteristics among the three genotypes at rs1254337 in our premature CAD patients. Furthermore, there was no association between this polymorphism and the severity of coronary lesions. Thus, we were not able to explain the mechanism responsible for the relationship between rs1254337 and premature CAD, despite the publication of a recent study that showed that earlier pubertal timing was associated with higher risks for angina, hypertension, and T2DM [24]. Therefore, it is still necessary to explore the pathophysiological mechanisms of pubertal transition-related gene polymorphisms involved in the progression of premature CAD as well as to establish reasonable prevention strategies to attenuate morbidity and mortality associated with premature CAD.

The present study has some strengths and limitations. First, our study sample is relatively small and this might have an impact on the statistical power. Second, this single-center, case-control study included only middle-aged subjects suspected of having premature CAD, so the participants did not represent the characteristics of the general population. The fact that all participants underwent elective CAG indicates that our results are reliable. Thirdly, since certain genotypes and alleles might be race-specific, it may not be possible extrapolate our results to other ethnic populations. Even so, the present study adds new information to this specific field and could serve as the basis for further studies.

## Conclusions

In a sample of subjects from China, our findings for the first time suggest that a genetic variant at rs1254337 may exert an influence on the development of premature CAD. However, this finding needs to be further verified by prospective studies in a larger population with diverse ethnicities to elucidate possible mechanisms. Furthermore, it is too early to regard the pubertal transition-related gene rs1254337 as a causal factor of premature CAD.
